# The End and the Beginning

**Published:** 1887-12-31

**Authors:** 


### WORDS OF CONSOLATION.

[Communications for this column should be addressed "Chaplain," care of the Editor of THE HOSPITAL, who will gratefully welcome any suggestions or inquiries from matrons, nurses, and the staff generally.]

### THE END AND THE BEGINNING.

THE end of the year brings with it an inevitable sadness. We cannot but think of disappointments and losses, of regrets that take the place of the hopes with which the year began. Above all, when the end of the year is marked by sickness and pain we are apt to lose heart, thinking sadly of our losses and not of our gain. And yet if we look over the past honestly we shall usually find that the one equals the other. For the rash expectations which have been disappointed we have received hopes less bright but more sure, and experience to guide our steps in future. Failure often proves our best teacher by showing us what to avoid; disappointment often brings in its train contentment with what we have; and every succeeding year adds to our store of knowledge, not merely comprehension of the things of this life, but understanding of God's purposes towards us. And every end is only a step from a new beginning, so close to each other are the cycles of life. The beginning of a new year has in it incalculable possibilities of improvement, of gain of every kind. Even when the tale of earthly years is told, when life itself is at an end, what is death but the step that leads to a new beginning—the beginning of an eternity whose possibilities of growth, intellectual and spiritual, and of purest happiness, our fondest conjectures can hardly hope to picture? If this be so—if the end of one experience or opportunity only marks the beginning of another—need we, in this upward-striving life of ours, ever despair? Surely not; we have only to look forward and go forward, sure that however dark the present is the future holds unguessed possibilities of improvement; sure, too, that if only our face be set in the right direction—towards God and truth—we cannot fail to reach higher things; and even if we sometimes stumble we shall fall, as has been said, "with our faces towards the light," and God Himself will stoop and raise us and guide our faltering steps.

